# Immune-mediated diseases after coronavirus disease 2019 vaccination: rare but important complication

**DOI:** 10.3325/cmj.2022.63.389

**Published:** 2022-08

**Authors:** Nikola Zagorec, Ivica Horvatić, Petar Šenjug, Matija Horaček, Danica Galešić Ljubanović, Krešimir Galešić

**Affiliations:** 1 Department of Nephrology and Dialysis, Dubrava University Hospital, Zagreb, Croatia; 2 School of Medicine, University of Zagreb, Zagreb, Croatia; 3 Department of Nephropathology and Electron Microscopy, Department of Pathology and Cytology, Dubrava University Hospital, Zagreb, Croatia

## Abstract

Since the beginning of mass vaccination against coronavirus disease 2019 (COVID-19), vaccine-linked immune-mediated diseases have been increasingly reported. The development of these diseases after COVID-19 vaccination may be attributed to the mechanisms of molecular mimicry and cross-reactivity between the viral spike protein and self-antigens. The most frequent vaccine-linked glomerular disease is immunoglobulin A nephropathy (IgAN). Cutaneous vasculitis has also been reported after COVID-19 vaccination. In both diseases, deposition of immune complexes activates the inflammatory response with end-organ damage. We report on a case of *de novo* IgAN in a young man and a case of severe cutaneous vasculitis in a 68-year-old woman, both after the second dose of Pfizer-BioNTech COVID-19 vaccine. Neither of the patients had a history of autoimmunity or adverse reactions to vaccines. The temporal association between vaccination and disease development in the absence of other possible intercurrent inciting events suggests a causal mechanism, although coincidental co-occurrence cannot be excluded. In both cases, immunosuppressive treatment was warranted to stop disease progression and to partially or completely resolve the disease. A timely reaction is needed if new-onset signs of an immune-mediated disease appear after vaccination.

Since the end of 2019 until July 2022, severe acute respiratory syndrome coronavirus 2 (SARS-CoV-2), causing coronavirus infectious disease 2019 (COVID-19), caused more than 6.35 million deaths worldwide (1). Although the vaccines against COVID-19 are generally well tolerated, and major side effects are uncommon, the number of vaccine-linked cases of *de novo* or relapsing glomerular and other immune-mediated diseases has been growing (2-6). Generally, the existing literature does not establish a causative link between vaccines and immune-mediated diseases, but temporal association and the absence of other potential causes put them in relation (7). The autoimmune disease most commonly related to vaccination, particularly influenza vaccination, is vasculitis (7,8). The most common type of vaccine-related vasculitis is immune-mediated cutaneous vasculitis, followed by IgA vasculitis and anti-neutrophilic cytoplasmic autoantibodies (ANCA)-associated vasculitis (7,9). Shortly after the introduction of mRNA COVID-19 vaccines (Moderna or Pfizer vaccine), there have been increasing reports of mRNA vaccines-related immune-mediated diseases, such as vasculitides (cutaneous, IgA vasculitis, ANCA-associated, large-vessel vasculitis) as well as glomerular and other autoimmune diseases (2-5,9-11). The most commonly reported glomerular disease related to COVID-19 vaccine is relapsing or *de novo* IgA nephropathy (IgAN), although its incidence is unknown (2,4,6). Herein, we report a case of *de novo* IgAN and a case of cutaneous vasculitis, both occurring after Pfizer-BioNTech COVID-19 vaccination. Reporting of such clinical cases is important to facilitate an appropriate recognition of complications of COVID-19 vaccines in daily clinical practice.

## CASE 1

**Figure 1:**
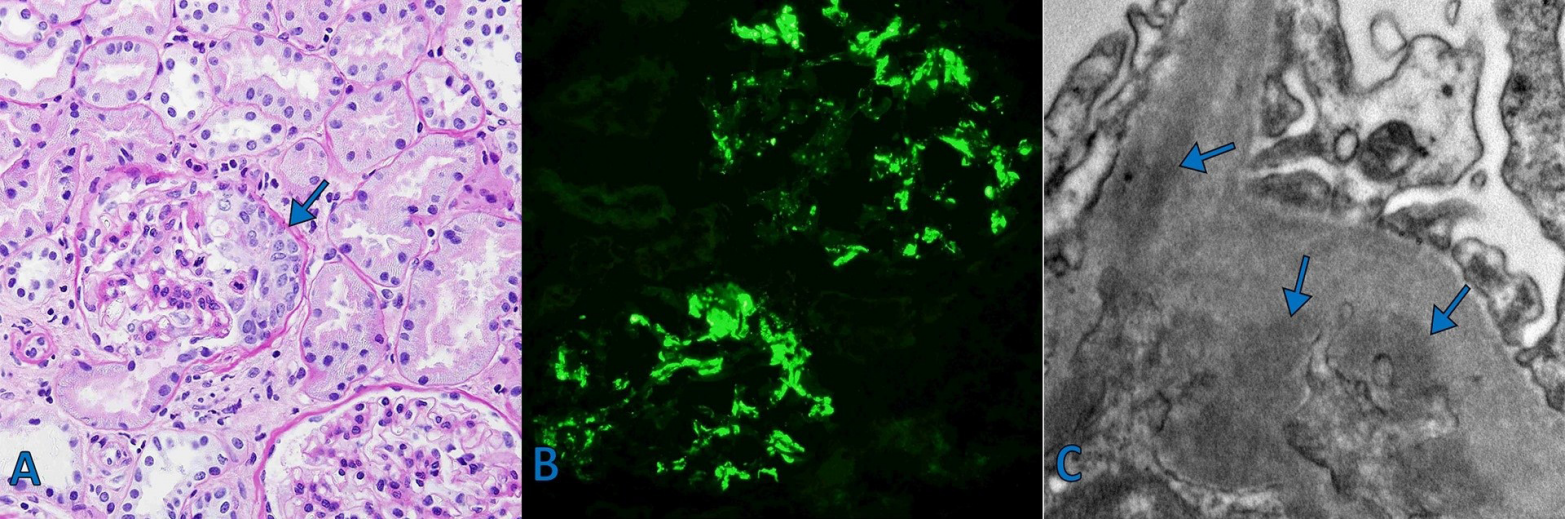
Case 1 – immunoglobulin (Ig) A nephropathy, kidney histology: (**A**) A glomerulus with cellular crescent (arrow) and mesangial hypercellularity on light microscopy, periodic acid Schiff stain, ×400. (**B**) IgA-positive glomeruli on immunofluorescence microscopy, ×400. (**C**) Mesangial dense deposits (arrows) on electron microscopy, ×30,000.

An otherwise healthy 26-year-old man presented in April 2021 with gross hematuria within 24 hours after the second dose of Pfizer-BioNTech COVID-19 vaccine. Six months earlier he had a SARS-CoV-2 infection presenting with fever and anosmia. The infection was confirmed by polymerase-chain reaction (PCR) of nasopharyngeal swab. He had no previous adverse reactions to vaccinations, including to the first dose of Pfizer vaccine, which he received three weeks before the second dose. Baseline serum creatinine and urinalysis were normal. Gross hematuria self-resolved within four days, but nephrotic-range proteinuria (3.5 g/d), microscopic hematuria (>40 erythrocytes per high-powered field), and mild creatininemia (peaked at 128 mmol/L) persisted. After the patient was referred to our center (100 days after the episode of gross hematuria), kidney biopsy revealed IgAN with crescents in 16% of glomeruli and mild tubulointerstitial scarring consistent with *de novo* IgAN (Figure 1). Relevant laboratory findings obtained during the diagnostic and follow-up period are shown in Table 1. Due to a high risk of disease progression, ramipril and methylprednisolone (MP) pulses (500 mg over three days) were introduced, followed by oral MP 0.8 mg/kg once a day. Two months later, in October 2021, the patient experienced partial disease remission with normalization of serum creatinine and significant reduction in proteinuria (Table 1).

**Table 1:** Relevant laboratory findings after initial symptoms onset (day 0) in both cases

Laboratory finding	Case 1			Case 2	
	Day 20	Day 100 (time of kidney biopsy)	Day 160 (60 days after MP* initiation)	Day 7	Day 65 (56 days after MP initiation)
Hemoglobin (g/L)	115	126	131	149	140
Leukocyte (x10^9^/L)	9	8.5	11.5	8.5	9.0
Serum creatinine (μmol/L)	128	102	94	77	51
Estimated glomerular filtration rate† (mL/min/1.73m^2^)	66	87	96	68	95
Total cholesterol (mmol/L)	5.3	-	5.4	3.5	-
Triglycerides (mmol/L)	1.3	-	2.8	0.9	-
Serum albumin (g/L)	39.6	42	43	36	40
C-reactive protein (mg/L)	5.8	3.0	-	86.3	9.1
C3 (g/L, ref. 0.9-1.8)	1.22	-	-	1.31	-
C4 (g/L, ref. 0.1-0.4)	0.39	-	-	0.37	-
Immunoglobulin G (g/L, ref. 7.0-16.0)	8.51	-	-	8.7	-
Immunoglobulin A (g/L, ref. 0.7-4.0)	2.38	-	-	1.7	-
Complete immunology and cryoglobulins	negative	-	-	negative	-
24-h proteinuria (g/d)	3.2	3.5	1.1	2.01	<0.3
Erythrocyturia (per high powered field)	>100	25	20	>40	<5

*MP – methylprednisolone.

†according to Chronic Kidney Disease Epidemiology Collaboration.

## CASE 2

**Figure 2:**
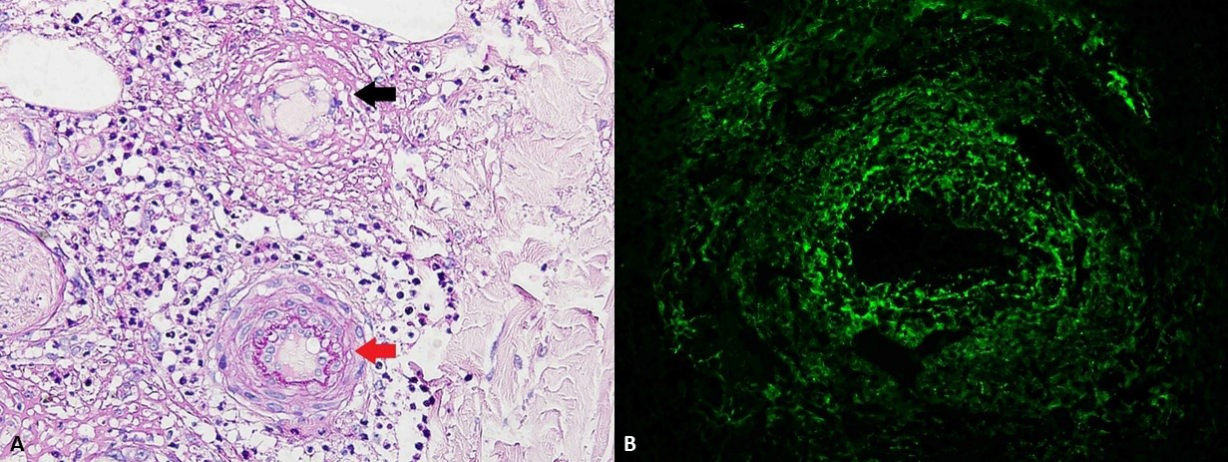
Case 2 – necrotizing vasculitis, skin histology. (**A**) Fibrinoid necrosis of vessel wall (black arrow). For comparison, normal artery is shown (red arrow). Periodic acid Schiff stain, ×400 (**B**) immunoglobulin-A positivity in an artery on immunofluorescence microscopy, ×400.

A 68-year-old woman presented in July 2021 to the emergency department with new-onset purpuric papules, multiple hemorrhagic vesicles and bullae of lower limbs, and purpuric papules of the dorsal side of the hands in symmetrical distribution. Hemorrhagic vesicles and bullae were surrounded by erythema and edema of the lower limbs. She had a history of arterial hypertension and depression. Her daily medications (without recent adaptations) included ramipril, verapamil, maprotiline, cinnarizine, and tramadol. At the admission, she was afebrile without any signs of infections. PCR to SARS-CoV-2 was negative. Six days before, she received the second dose of Pfizer BioNTech COVID-19 vaccine. She had no previous adverse reactions to vaccinations or a history of autoimmune diseases. Table 1 shows the results of the initial laboratory workup. Deep venous thrombosis and pulmonary embolism, suspected due to elevated D-dimers and lower limb edema detected on admission and further clinical workup, were excluded based on color Doppler ultrasound and CT pulmonary angiography. Complete immunological findings (antinuclear antibodies, anti-dsDNA, ANCA, rheumatoid factor, anticardiolipin antibodies), cryoglobulins, serum protein immunofixation, and serology for HIV and hepatitis viruses were negative. The fecal occult blood test was negative. Skin biopsy revealed severe necrotizing vasculitis of small and medium-sized arteries with fibrinoid necrosis and mixed neutrophil-mononuclear infiltrate. Direct immunofluorescence showed positive staining for IgA, IgM, and C3 in the arterial wall (Figure 2). Kidney biopsy showed only mild nephroangiosclerosis with no glomerular involvement. Angiography of the aorta with visceral branches did not reveal any aneurysms or signs of systemic vasculitis. She was started empirically on MP (1 mg/kg) intravenously on day 3 after admission. Proteinuria and erythrocyturia completely resolved during the hospital stay. After discharge, oral MP (32 mg once a day) was continued, but rapid tapering of MP dose was necessary due to complications of steroids therapy (hyperglycemia, enterocolitis caused by *Clostridioides difficile*). Within two months, skin lesions almost completely resolved.

## DISCUSSION

We report on two cases of *de novo* immune-mediated diseases after COVID-19 vaccine. Although the appearance of gross hematuria in case 1 shortly after the second dose of vaccine may suggest a causal mechanism, we cannot exclude a coincidental co-occurrence. Klomjit et al (2) published a largest series of cases of *de novo* or relapsing glomerular disease related to mRNA vaccines, among which IgAN was the most common. The immune response to COVID-19 vaccine may unmask preexisting undiagnosed IgAN in some cases (2,4,6), but in our case, normal urine sediment and serum creatinine before vaccination and only mild tubulointerstitial scarring on the kidney biopsy specimen indicate a *de novo* disease. Furthermore, kidney disorders in case 1 did not resolve spontaneously, warranting immunosuppressive treatment to prevent disease progression. The appearance of IgAN symptoms one day after the second dose of vaccine might be explained by the formation and precipitation of immune complexes composed of aberrant IgA1 and antiglycan antibodies. Mucosal infections such as COVID-19 enhance the production of interleukin-1, which stimulates the production of poorly galactosylated IgA1 and the formation of aberrant IgA1, contributing to the development of IgAN (12). However, we cannot exclude the participation of innate immune response, especially if we consider the short period between viral antigen exposure and the onset of symptoms. The following mechanisms of innate immunity are included in the pathogenesis of IgAN: complement system activation by IgA1 via lectin and alternative pathway, activation of transferrin receptors expressed on mesangial cells, and activation of other receptors, such as soluble CD89, which promotes inflammatory response in the kidney (12,13).

Cutaneous vasculitis is precipitated by infections, medications, and vaccines such as those against hepatitis A and B, influenza, Bacillus Calmette-Guérin, and human papillomavirus (7). In our case, an obvious temporal association between vaccination and vasculitis development without any intercurrent inciting events indicates vaccine-linked vasculitis, although a coincidental co-occurrence cannot be excluded. A growing number of similar reports of vasculitis after COVID-19 vaccination and COVID-19 infection further supports the correlation between the vaccine and cutaneous vasculitis (3,5,9-11,14). mRNA COVID-19 vaccines induce robust immune response (B/T cell activation and antibodies formation) mediated by the viral spike protein. This can result in an immune complexes formation and their deposition in vessel walls, causing vasculitis. Possible underlying mechanisms of such a reaction are molecular mimicry and cross-reactivity between the viral and host endothelial antigens (7,15). The involvement of medium-sized arteries in the case 2 raised the suspicion of a systemic character of vasculitis, but extensive diagnostic workup, including kidney biopsy, did not detect any signs of systemic involvement due to vasculitis.

Although the benefits of COVID-19 vaccination strongly outweigh the risks (16), rare immune-mediated adverse events in susceptible individuals with maladaptive immune response are possible. They should not lead to vaccine hesitation because these patients are few compared with those who have safely received the vaccine (4). These cases emphasize the need for vigilance in patients presenting with new-onset skin lesions and urine abnormalities after COVID-19 vaccination. The correlation between COVID-19 vaccines and immune-mediated diseases remains to be elucidated.

Declaration of authorship NZ conceived and designed the study and acquired the clinical data. All authors analyzed and interpreted the data. NZ drafted the manuscript. DGLJ, PS and MH prepared histological images. IH, PS, MH, DGLJ and KG critically revised the manuscript for important intellectual content. All authors gave approval of the version to be submitted and agree to be accountable for all aspects of the work.
